# Melatonin Attenuates *Ferritinophagy*/Ferroptosis by Acting on Autophagy in the Liver of an Autistic Mouse Model BTBR T^+^Itpr3^tf^/J

**DOI:** 10.3390/ijms252312598

**Published:** 2024-11-23

**Authors:** Giorgia Cominelli, Claudio Lonati, Daniela Pinto, Fabio Rinaldi, Caterina Franco, Gaia Favero, Rita Rezzani

**Affiliations:** 1 Anatomy and Physiopathology Division, Department of Clinical and Experimental Sciences, University of Brescia, 25123 Brescia, Italy; giorgia.cominelli@unibs.it (G.C.); claudio.lonati@unibs.it (C.L.); gaia.favero@unibs.it (G.F.); 2 Italian Society for the Study of Orofacial Pain (Società Italiana Studio Dolore Orofacciale—SISDO), 25123 Brescia, Italy; 3 Human Microbiome Advanced Project Institute, 20129 Milan, Italy; 4 Interdepartmental University Center of Research Adaption and Regeneration of Tissues and Organs-(ARTO), University of Brescia, 25123 Brescia, Italy

**Keywords:** melatonin, autism spectrum disorder, liver injury, Mallory–Denk bodies, autophagy, *ferritinophagy*, ferroptosis

## Abstract

Autism spectrum disorders (ASDs) are a pool of neurodevelopment disorders in which social impairment is the main symptom. Presently, there are no definitive medications to cure the symptoms but the therapeutic strategies that are taken ameliorate them. The purpose of this study was to investigate the effects of melatonin (MLT) in treating ASDs using an autistic mouse model BTBR T^+^Itpr3^tf^/J (BTBR). We evaluated the hepatic cytoarchitecture and some markers of autophagy, *ferritinophagy*/ferroptosis, in BTBR mice treated and not-treated with MLT. The hepatic morphology and the autophagy and *ferritinophagy*/ferroptosis pathways were analyzed by histological, immunohistochemical, and Western blotting techniques. We studied p62 and microtubule-associated protein 1 light chain 3 B (LC3B) for evaluating the autophagy; nuclear receptor co-activator 4 (NCOA4) and long-chain-coenzyme synthase (ACSL4) for monitoring *ferritinophagy*/ferroptosis. The liver of BTBR mice revealed that the hepatocytes showed many cytoplasmic inclusions recognized as Mallory–Denk bodies (MDBs); the expression and levels of p62 and LC3B were downregulated, whereas ACSL4 and NCOA4 were upregulated, as compared to control animals. MLT administration to BTBR mice ameliorated liver damage and reduced the impairment of autophagy and *ferritinophagy*/ferroptosis. In conclusion, we observed that MLT alleviates liver damage in BTBR mice by improving the degradation of intracellular MDBs, promoting autophagy, and suppressing *ferritinophagy*/ferroptosis.

## 1. Introduction

Autism spectrum disorders (ASDs), a series of heterogeneous neurodevelopmental disorders, are characterized by stereotyped behavior and deficits in communication, interaction, and perception [1,2,3]. ASDs begin in childhood or earlier and lasts throughout the individual’s life [4]; the disorder frequently has an impact on personal, social, school, and work life [5]. ASDs are so-called male-predominant conditions, as shown by the meta-analytical prevalence rates with an overall male-to-female ratio of 4.20:1 (95% CI 3.84–4.60) [6,7,8,9,10]. The source of this marked difference remains a mystery; its basis is likely to be multifactorial but is poorly understood. Few of the more than 100% thus far identified risk genes reside on sex chromosomes. Baron-Cohen has proposed the “extreme male brain theory” of ASDs, attributing sex differences to testosterone levels [10,11]. This is plausible because multiple lines of evidence suggest dysregulated steroidogenesis, but testosterone levels are unlikely to fully explain the male/female discrepancy [10,12,13].

In recent years, many studies have suggested a link between ASDs and multiple environmental, as well as genetic, factors without defining the specific pathogenesis [2]. Patients with ASDs have a high prevalence of functional gastrointestinal disorders (FGIDs) [5,14], now indicated as disorders of gut–brain interaction (DGBIs) [15]. These observations have led to an increase in research to identify differences in the gastrointestinal (GI) system in these neurodevelopmental disorders, with a particular interest in the microbiota. Most importantly, it has been shown that the gut flora is able to adjust the intestinal metabolites, and this could have an impact on behavior, interest, and sleep patterns in children with ASDs. Specifically, GI tract dysfunction in autistic children is due to mitochondrial imbalance, oxidative stress, and inflammation [16,17,18,19,20].

The communication system between the central and the enteric nervous systems, also known as the gut–brain axis, plays an important role in maintaining metabolic homeostasis and developing cognitive functions. In fact, several studies show the presence of metabolic and GI diseases in patients with ASDs [21,22,23,24]. Within the gut–brain axis lies the liver, an organ responsible for performing essential metabolic functions and maintaining the balance of the entire body. By creating a link between the central nervous system and peripheral systems, the liver emerges as an interesting target for studying the etiology of ASDs and developing new therapeutic approaches [25]. 

An autistic mouse model, BTBR T^+^Itpr3^tf^/J (BTBR), can be used as a translation tool for evaluating potential therapies for ASDs and related comorbidities because these animals exhibit the behavioral, electrical, and molecular features of ASDs [19,26,27,28].

Trinchese et al. [29] published the results of a pioneering study showing the involvement of hepatic mitochondrial functions in BTBR mice. The study shows that these animals exhibit increased inflammation and oxidative stress related to mitochondrial dysfunction, steatotic hepatocytes, and mitochondrial fission [16]. Recently, our research group found that BTBR mice show many changes in liver morphology and an increase in the expression of ferroptosis pathway markers, indicating that liver damage is also implicated in ASDs [3]. Ferroptosis is a form of non-apoptotic programmed cell death (PCD) characterized by a lethal level of iron-dependent accumulation of lipid hydroperoxides [30,31]. It plays an important role in the development of liver diseases, as excessive hepatocytes death can lead to severe liver damage [32]. Previous studies reported that ferroptosis is correlated to the etiology and pathological mechanisms of ASDs [33,34,35]. 

Autophagy is a conserved intracellular catabolic PCD process that delivers cellular components, such as lipid droplets, protein aggregates, and organelles, to the lysosomes to be degraded by the formation of autophagosomes [36,37]. At a moderate level, autophagy aims to maintain intracellular homeostasis and increase the survival rate of cells under stress [38]. In contrast, impaired or excessive autophagy can lead to cell death due to excessive degradation or metabolic crises [39]. 

An increasing body of evidence has revealed a link between autophagy and ferroptosis, suggesting that autophagy is involved in the regulation of iron-dependent lipid peroxidation of the ferroptosis pathway [38,40,41]. It has been previously shown that abnormal ferritin aggregation and excessive degradation by autophagy leads to intracellular iron overload, which ultimately triggers lipid reactive oxygen species (ROS) accumulation [34,42,43]. Ferritin is a cellular iron reserve protein, and its degradation results in increased levels of labile iron. Hou et al. [44] found that excess autophagy leads to iron accumulation and lipid peroxidation through degradation of anti-ferroptosis regulators. Instead, Wang et al. [45] showed that decreased autophagy further encourages iron-excess-induced ferroptosis. Despite this, a comprehensive and mechanistic understanding of the role of autophagy on ferroptosis or vice versa remains lacking and in this field of research is included our study. 

p62 is an autophagy substrate that also serves as a selective autophagy receptor for degrading protein aggregates and damaged organelle protein [46]; in terms of physiological conditions, it plays an important role in maintaining metabolic homeostasis [47]. The ability of p62 to act as an autophagy receptor is based on specific interactions with different autophagy-related proteins, such as microtubule-associated protein 1 light chain 3 B (LC3B). LC3B is a component of the autophagosome membrane, and its interaction with p62 promotes autophagy [48,49]. 

As reported by Gao et al. [50], impaired autophagy also stimulates the degradation of ferritin through the nuclear receptor co-activator 4 (NCOA4). Such NCOA4-mediated autophagic degradation of ferritin is termed *ferritinophagy*. NCOA4 governs *ferritinophagy,* which mediates ferritin aggregation and translocation to lysosomes for degradation and subsequent intracellular iron overload [34,51]. 

Therefore, NCOA4-mediated *ferritinophagy* plays an important role in the promotion of ferroptosis as it leads to the accumulation of iron and ROS [43,52,53]. Consequently, *ferritinophagy* contributes to the initiation of ferroptosis, which is, in turn, related to the etiology and pathological mechanism of ASDs [33,34,35]. A plausible strategy for ASDs treatment might be the inhibition of *ferritinophagy*, which could result in autistic symptom alleviation via ferroptosis suppression.

Long-chain-coenzyme (CoA) synthase (ACSL4) is involved in the regulation of arachidonic acid and eicosapentaenoic acid and plays a role in the activation of ferroptosis through the initiation of phospholipid peroxidation. Its overexpression alters cellular lipid composition, increasing the susceptibility of cells to ferroptosis [54,55]. 

Melatonin (N-acetyl-5-methoxyltryptamine) is synthesized in the pineal gland, digestive organs, and other extra-pineal organs and plays an important role in various biological processes, including sleep and aging and cardiovascular, renal, and hepatic diseases [56,57,58]. Many studies have reported the benefits of MLT administration, such as its antioxidant, analgesic, and anxiolytic effects, which slow down aging-related diseases [59].

In our previous experimental study, we reported that MLT, an amphiphilic indoleamine, acts in inhibiting inflammation, oxidative stress, and ferroptosis and can counteract or modulate hepatic ASDs-related alterations [3,33].

The regulatory mechanisms of autophagy and *ferritinophagy*/ferroptosis following MLT administration in BTBR mice need further investigation. Therefore, the present study was aimed at exploring in more detail the effect of MLT on liver damage in BTBR mice, considering the relationship between autophagy and *ferritinophagy*/ferroptosis. 

First, we investigated the cytoarchitecture of liver parenchyma in BTBR mice and MLT-treated BTBR mice; then, we applied immunohistochemical and biochemical analysis to assess certain proteins involved in autophagy, *ferritinophagy,* and ferroptosis pathways. In particular, we evaluated p62, a substrate for autophagy and a critical component of cytoplasmic inclusions known as Mallory–Denk bodies (MDBs) [60,61]; LC3B, which contributes to autophagosome formation by stimulating the autophagic process [62]; NCOA4, an important biomarker of *ferritinophagy*; ACSL4, a biomarker and contributor to ferroptosis [54,55]. Furthermore, we evaluated the possible protective effect of MLT against ASDs–liver comorbidity by assessing its potential mechanism(s) of action on the markers reported above. 

In conclusion, these findings could be useful for the evaluation of ASDs, in view of the fact that autophagy and *ferritinophagy*/ferroptosis may be clinical targets in these disorders and pointing out that MLT administration can open up new possibilities for the treatment of ASDs comorbidities. 

## 2. Results

The vehicle of MLT did not interfere with the effects of MLT; therefore, BTBR mice and CTR mice treated with MLT vehicle were defined as “BTBR mice” and “CTR mice”, respectively. Furthermore, CTR mice treated or not with MLT displayed similar “normal” behavioral and morphological data; so, in the present study, they were defined generically as a unique group “CTR mice”.

### 2.1. Effects of MLT on Hepatic Morphopathological Changes 

**Figure 1:**
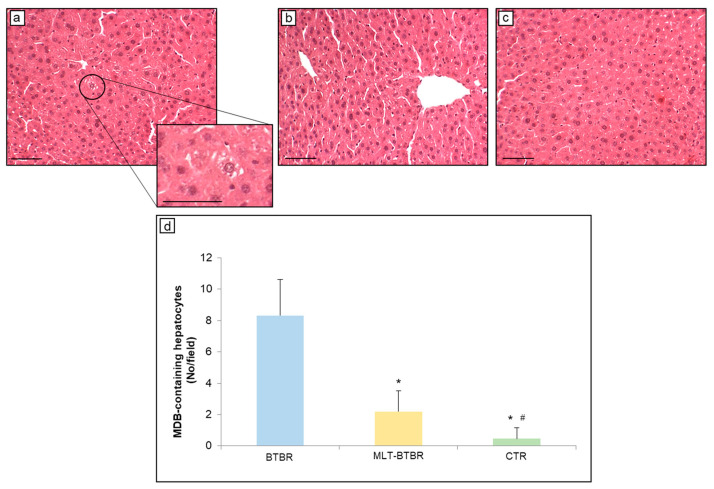
Morphopathological evaluation of hepatocytes containing MDBs. Representative photomicrographs of hematoxylin–eosin staining of (**a**) BTBR mice, (**b**) MLT-treated BTBR mice, and (**c**) CTR mice. Original magnification: 200×; insert: 400×; bars = 50 µm. (**d**) Quantitative analysis of MDB-containing hepatocytes. * *p* < 0.05 vs. BTBR mice; # *p* < 0.05 vs. MLT-BTBR mice.

The hepatic parenchyma of BTBR mice was preserved even in the case of very evident hepatocellular ballooning, as already reported [3]. Interestingly, we noted that a moderate number of enlarged (ballooned) hepatocytes showed inclusions defined as MDBs (Figure 1a). MDBs were seen as irregularly shaped cytoplasmic inclusions, and, sometimes, they appeared as numerous small globular structures distributed throughout the hepatocyte cytoplasm (Figure 1a, insert). Figure 1b,c show a well-preserved liver parenchyma morphology of MLT-treated BTBR mice and CTR mice, respectively. The quantitative analyses confirmed the presence of a significative number of MDB-containing hepatocytes in BTBR mice as compared to BTBR mice treated with MLT or CTR mice (Figure 1d). In particular, the number of hepatocytes with MDBs in MLT-treated BTBR mice was visibly reduced with respect to BTBR mice, although it was slightly higher than in CTR mice. 

### 2.2. Effects of MLT on Hepatic p62 and LC3B

The hepatic expression and levels of p62 and LC3B were examined in all experimental groups; for both markers, it was found that hepatic expression and levels were lower in BTBR mice as compared with MLT-treated BTBR mice and CTR mice. 

**Figure 2:**
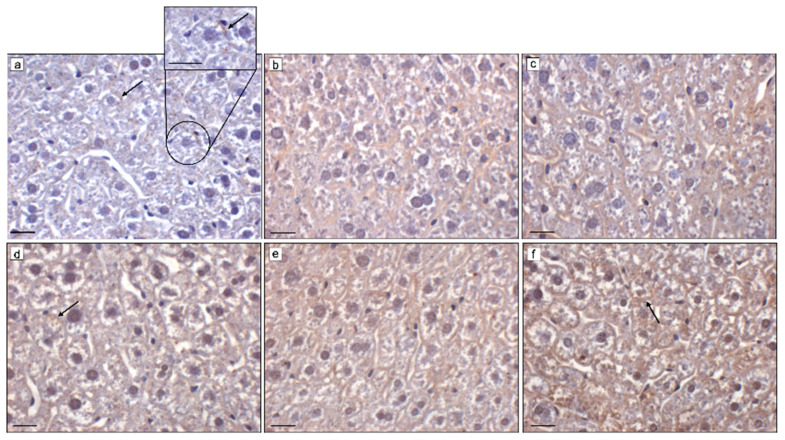
Hepatic p62 and LC3B immunohistochemical evaluations. Representative photomicrographs of liver p62 (**a**–**c**) and LC3B (**d**–**f**); immunostainings of (**a**,**d**) BTBR mice, (**b**,**e**) BTBR mice treated with MLT, and (**c**,**f**) CTR mice. Black arrows indicate positivity in MDBs. Original magnification: 400×; insert: 1000× (**b**); bars = 20 µm.

The immunopositivity of p62 was very weak/weak evident in the cytoplasm of hepatocytes and sometimes in the MDBs of BTBR mice (Figure 2a, insert), while no positivity was observed at nuclear level (Figure 2a). In MLT-treated BTBR mice, p62 showed moderate positivity in the cytoplasm of hepatocytes and very weak/absent immunopositivity in MDBs and nuclei; similar immunopositivity was also found in the hepatocytes of CTR mice (Figure 2b,c). The semiquantitative analysis of p62 immunopositivity confirmed the morphological evaluation reported above (Table 1). 

LC3B immunopositivity in BTBR mice was weak in the cytoplasm of hepatocytes and moderate/strong in the nuclei, while MDBs were very weak (Figure 2d). In MLT-treated BTBR mice, LC3B expression was moderate and mainly localized in the cytoplasm of hepatocytes, and, differently from BTBR mice, the nuclei and the MDBs presented very weak LC3B immunopositivity (Figure 2e). In CTR animals, LC3B expression was moderate in the cytoplasm of hepatocytes and very weak in the nuclei and in the MDBs (Figure 2f). The semiquantitative analysis of LC3B immunopositivity confirmed morphological evaluation reported above (Table 1).

**Figure 3:**
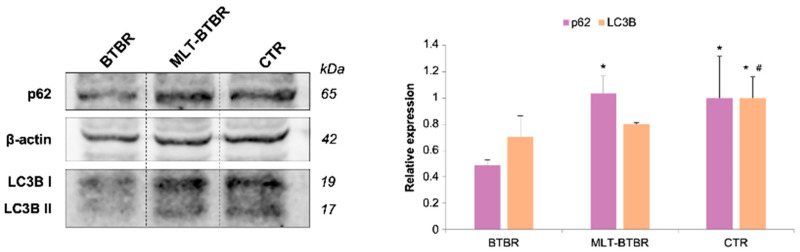
Hepatic p62 and LC3B Western blotting. Representative immunoblots of p62, LC3B I, and LC3B II of total liver samples from BTBR mice, BTBR mice treated with MLT, and CTR mice. β-actin was used as loading control. Relative expression quantification of Western blotting for p62 and LC3B. * *p* < 0.05 vs. BTBR mice; # *p* < 0.05 vs. MLT-BTBR mice.

The immunohistochemical data on p62 shown above were also confirmed using a Western blotting evaluation, as reported in Figure 3. The Western blotting evaluation also confirmed the LC3B immunohistochemical data reported above. In particular, LC3B immunoblotting displayed two distinct bands at approximately 19 and 17 kDa, representing the cytosolic form LC3B I and the membrane form LC3B II, which is conjugated with phosphatidylethanolamine and is present on autophagosomes. The results for p62 bands were analyzed quantitatively using β-actin as normalizing protein, while the results for LC3B bands were analyzed quantitatively by calculating the ratio between the two forms of the protein (LC3BII/I) (Figure 3). Notably, BTBR mice showed low levels of both p62 and LC3BII/I ratio compared to CTR mice, which displayed a higher p62 and LC3BII/I ratio. MLT-treated BTBR mice as compared to BTBR mice showed a significant increase in p62 and a slight increase in LC3BII/I ratio. Interestingly, an increase in the LC3BII/I ratio indicates a physiological autophagic process with a correct number of autophagosomes that are ready to fuse with the lysosomes.

### 2.3. Effects of MLT on Hepatic NCOA4 and ACSL4 

**Figure 4:**
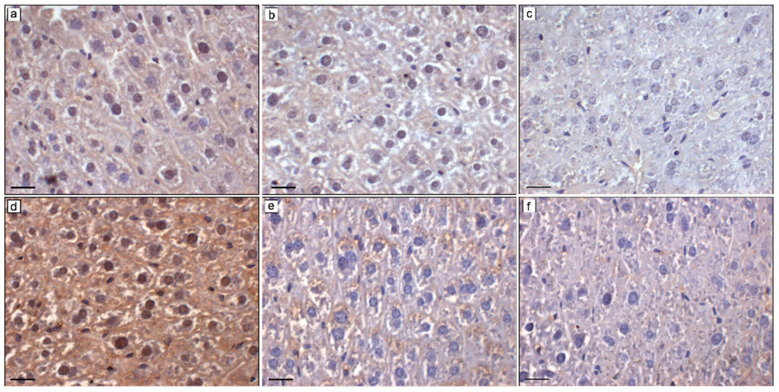
Hepatic NCOA4 and ACSL4 immunohistochemical evaluations. Representative photomicrographs of liver NCOA4 (**a**–**c**) and ACSL4 (**d**–**f**) immunostaining of (**a**,**d**) BTBR mice, (**b**,**e**) BTBR mice treated with MLT, and (**c**,**f**) CTR mice. Original magnification: 400×; bars = 20 µm.

The immunopositivity for NCOA4 in the hepatocytes of BTBR mice was moderate at the cytoplasmic and nuclear levels and very weak/weak in MDBs (Figure 4a). In MLT-treated BTBR mice, the hepatocyte cytoplasm showed a weak/very weak positivity for NCOA4, while the nuclei and MDBs displayed very weak and absent immunopositivity for NCOA4, respectively (Figure 4b). Very weak positivity was also observed in the cytoplasm of hepatocytes of CTR mice, while the MDBs and nuclei showed no NCOA4 immunopositivity (Figure 4c). The semiquantitative analysis of NCOA4 immunopositivity confirmed the morphological evaluation reported above (Table 2).

The immunopositivity for ACSL4 was strong in the hepatocyte cytoplasm and nuclei and weak/moderate in the MDBs of BTBR mice (Figure 4d). In MLT-treated BTBR mice, ACSL4 showed weak/moderate positivity in the hepatocyte cytoplasm (Figure 4e), and CTR mice presented very weak ACSL4 immunopositivity in the hepatocyte cytoplasm (Figure 4f). No positivity was observed in the hepatocyte nuclei in both MLT-treated BTBR mice and in CTR mice. Very weak/absent positivity was observed in the MDBs of MLT-treated BTBR mice, while the MDBs showed no ACSL4 immunopositivity in CTR mice (Figure 4e,f). The semiquantitative analysis of ACSL4 immunopositivity confirmed the morphological evaluation reported above (Table 2).

## 3. Discussion

ASDs are commonly recognized for its social deficits and stereotyped behaviors [34]; its etiology is unknown despite the fact that multifactorial disorders with strong genetic components have been found [34,63].

Several studies have focused on nervous system alterations, but new data have shown that GI problems are commonly found in children with ASDs [64,65,66]. As described above, the findings of Trinchese et al. [3] and of our research group [16] showed several alterations in the liver of BTBR mice. We found that the liver of BTBR mice showed some defects in the organogenesis, and these defects are comparable to those observed in hippocampal neurogenesis in various ASDs models and ASDs patients [3,67,68]. Moreover, we showed that MLT administration in BTBR mice has positive effects on hepatic cytoarchitecture and metabolic functions, including anti-ferroptosis mechanisms.

Following these results, we explored in greater detail: the cytoarchitecture of liver parenchyma in BTBR mice treated and not-treated with MLT; the pathway by which MLT can ameliorate liver damage in autistic mice. For this purpose, we evaluated certain proteins involved in the autophagy and *ferritinophagy*/ferroptosis mechanisms. 

Through morphological evaluation, we found several cytoplasmic inclusions in the hepatocytes of BTBR mice, and we identified these inclusions as MDBs. It is known that MDBs are associated with a variety of liver diseases, including non-alcoholic fatty liver disease (NAFLD), as reported by Zatloukal et al. [69]. MDBs are made up of many proteins involved in the protein degradation machinery, such as keratin, ubiquitin, and p62. It is suggested that MDBs are most found in the liver, neural tissues, and muscle, and this could represent the interaction of different variables in these organs, including the extent of protein synthesis and turnover, oxidative and other stresses, and the level of autophagy. In particular, autophagy has been shown to contribute to preventing the formation of MDBs and may even promote their regression when activated [69,70]. MDBs are preferentially observed in enlarged ballooned hepatocytes, and, as we demonstrated above, the hepatocytes of BTBR mice presented significant microvescicular steatosis (hepatocellular ballooning) [3]. In addition, sex also plays a crucial role in MDB formation, since they are more evident in male mice than in female mice [52]. All these findings concur with our results showing that MDBs are more evident in BTBR mice in which protein degradation is impaired and that, in these male mice, they are found in greater quantities. The latter finding supports the findings on ASDs-related injuries, which affect mostly male children [71]. The reported results are obtained on male mice and so are generalized to the prevalence of ASDs patients. This choice was also made because female BTBR mice do not exhibit the behavioral inflexibility and lower order of restricted repetitive behaviors commonly observed in male BTBR mice [72]. Rather than searching for the causes of heightened risk in males, one might look, instead, for female protective factors. Girls who are diagnosed with ASDs carry significantly more genetic load than boys [10,73], suggesting adaptive mechanisms, such as the ability to effectively mask ASDs symptoms [10,74]. Since diagnostic criteria are based on the much more prevalent male presentation, it is likely that girls, who present somewhat differently, are underdiagnosed [10,75]. It is necessary to conduct further research to experimentally validate whether the mechanisms described here also occur in female patients. 

The present immunohistochemical and biochemical results showed that p62 and LC3BII/I ratio are downregulated, while NCOA4 and ACSL4 are upregulated, in the liver of BTBR mice, as compared to CTR mice. 

The p62 protein is a selective autophagy receptor that recruits and distributes intracellular substrates for bulk clearance through the lysosomal autophagy pathway [61,76,77]. When the expression and levels of p62 are modulated with respect to physiological homeostasis, autophagic flux is impaired, inducing the accumulation of intracellular components [77,78,79,80]. The perturbation of p62 activity has been frequently associated with the pathogenesis of many liver diseases, as reported by Tan et al. [61]. Our immunohistochemical and biochemical results concur with these data, since the immunopositivity and levels of p62 were weakly expressed in BTBR mice with respect to CTR mice, indicating an impairment in the autophagic process. Regarding liver morphology in BTBR mice and considering the reduced expression and low levels of p62, we hypothesized that, when the expression and levels of p62 are physiologically incorrect, it is responsible for the formation of MDBs and impairs their degradation. Based on these considerations, we suggest that, by mediating the balance between MDBs formation and degradation, p62 may affect the number of these inclusions in the hepatocytes of BTBR mice, as shown in alcohol-associated liver disease (ALD) [52]. 

As described above, we found reduced expression and levels of LC3B in the liver of BTBR mice, as compared with CTR mice; it is known that LC3B is important for autophagosome formation and, like p62, for autophagic flux [81]. The interaction between p62 and LC3B is necessary for the degradation of p62-positive bodies (MDBs) and for physiological autophagy [82]. The main constituents of MDBs are p62, keratins, ubiquitin, and chaperones; actually, it is not clear whether abnormal proteins are sequestered in a random manner or selectively by autophagy [83]. One possible explanation may be that p62 binds both ubiquitin and LC3B. Therefore, some mechanisms related to hepatocyte autophagy remain to be explored. 

Interestingly, immunoblotting found that the endogenous LC3B is expressed in two bands with different molecular weights, as reported by several authors [77,82]. One of these bands is LC3BI, which is the precursor to the band LC3BII, the latter being important for the biogenesis and/or closure of autophagosome membranes in the late stages of autophagy [76,84,85,86]. In BTBR mice, the LC3BII/I ratio was reduced compared to CTR mice. The reduction in the ratio in BTBR mice could indicate impaired autophagy, which is also supported by the low expression and levels of p62. Indeed, p62 is an autophagy substrate that combines with LC3BII to facilitate autophagosome degradation [87,88]. Therefore, we hypothesize that low expression and levels of both p62 and the LC3BII/I ratio indicate that hepatocytes fail to produce autophagosomes ready for degradation.

Moderate autophagy functions as a pro-survival mechanism when cells encounter stresses such as nutrient starvation and hypoxia, as well as range of anti-cancer therapies [50]. The impairment of autophagy promotes ferroptosis, which is characterized by enhanced bioavailability of intracellular iron ions, as previously reported by Wang et al. [45]. Recently, several reports have suggested that the impairment of autophagy leads to increased expression and levels of NCOA4, a transport receptor that induces *ferritinophagy,* and ACSL4, a vital initiator of ferroptosis without enough glutathione peroxidase 4 (GPX4) [52,54,89]. The presence of cellular iron is crucial for maintaining metabolic processes, but excessive ferrous iron can lead to oxidative damage and cell death [90]. *Ferritinophagy*, a selective form of autophagy, is mediated by NCOA4 and involves the excessive degradation of ferritin, releasing free iron within cells through the autophagy pathway [34,91,92].

NCOA4 and ACSL4 immunopositivity and levels were higher in the liver of BTBR mice, as compared to CTR mice. NCOA4 governs *ferritinophagy*, a phenomenon characterized by the autophagic degradation of ferritin to release intracellular free iron [34]; ferritin, which is a free iron storage, is composed of ferritin heavy chain 1 (FTH1) and ferritin light chain (FTL) [93]. NCOA4 interacts with FTH1 to mediate the transport of ferritin to lysosomes, inducing the formation of autophagosomes and the release of iron ions for the body’s metabolic needs [86,94]. When this mechanism is overactivated, the intracellular iron overloads, and this pathway contributes to the initiation of ferroptosis [95].

Impaired autophagy is a common feature found in association with ASDs [96,97].

ACSL4 modifies the phospholipid compositions of cell membranes and is involved in lipid metabolism. It catalyzes the formation of arachidonoyl-coenzyme A (AA-COA), which accumulates on the cellular membrane, triggering ferroptotic membrane ruptures [54,89]. Previously, we demonstrated that ferroptosis is a mechanism involved in the liver damage of BTBR mice; in this study, we demonstrated that the overexpression of NCOA4, as a marker of *ferritinophagy,* could increase susceptibility to ferroptosis with increased expression and levels of ACSL4. We observed increased expression and levels of both NCOA4 and ACSL4 in BTBR mice with respect to CTR mice. These findings confirm the mechanism described above.

Mounting evidence suggests that the administration of antioxidant substances, such as MLT, can ameliorate the damage in BTBR mice [3,18,19,98,99].

As described earlier, we further investigated the MLT mechanisms of action in BTBR mice. We found a reduced number of hepatocytes with intracellular MDBs, which could indicate a reduction in oxidative stress and inflammation with degradation of misfolded proteins, as reported by Zatloukal et al. [69]; a moderate and weak increase in p62 and LC3B, respectively, as important markers of efficient physiological autophagy; a decrease in ACSL4 and NCOA4 as possible targets for reduced *ferritinophagy*/ferroptosis.

Regarding the LC3B results, in BTBR mice, we found a notable and slight reduction in the LC3BII/I ratio compared to CTR mice and MLT-treated BTBR mice, respectively. If we consider the results obtained for p62 and LC3B together, a self-friendly pathway seems to change or improve in MLT-treated BTBR mice, as the expression and levels of p62 increase to a greater extent than the LC3BII/I ratio. Moreover, we found a strong LC3B immunopositivity in the hepatocyte nuclei of BTBR mice as compared to MLT-treated BTBR mice and CTR mice. Moreover, we found a strong LC3B immunopositivity in the hepatocyte nuclei of BTBR mice as compared to MLT-treated BTBR mice and CTR mice. It is known that LC3B must be localized at the cytosolic level to play its role in the autophagic process [100,101]; however, the nuclear fraction of LC3B can serve as a reservoir of cytosolic LC3B [102]. To mobilize this nuclear stock, LC3B must be deacetylated by Sirtuin-1 (Sirt-1) to translocate into the cytoplasm and interact with autophagic effectors [103,104,105]. Considering the impact of various stress conditions on Sirt-1 activity [106,107,108,109], we hypothesize that a decrease in Sirt-1 activity in the liver of BTBR mice could explain the reduction in LC3B mobilization and the impairment in the autophagic flux we observed. Furthermore, as reported by Brischetto et al. [110], the accumulation of LC3B at the nuclear level could be explained by a stress-induced Nf-kB activity, which is also associated with autophagy suppression. Both Sirt-1 and Nf-kB would need in-depth investigations in order to confirm the hypotheses made regarding the nuclear localization of LC3B and its role in autophagy.

The other interesting finding of this study was that the markers of ferroptosis increase in the expression and levels in MLT-treated BTBR mice. Therefore, we hypothesize that downregulation of ferroptosis is important to maintain the hepatocyte survival process and prevent liver damage, as reported by Yu and Wang [32].

## 4. Materials and Methods

### 4.1. Experimental Groups 

The experimental groups were organized in agreement with our previous works referring to this experimental project [18,19,99,111].

A total of 20 male BTBR T^+^Itpr3^tf^/J mice (BTBR) (JAXTM Mice Strain; The Jackson Laboratory, Bar Harbor, ME, USA) as transgenic animal model of ASDs and 20 C57BL6/J mice (JAXTM Mice Strain; The Jackson Laboratory, Bar Harbor, ME, USA) as healthy CTR mice were housed in cages (two/three animals/cage), with food and water ad libitum, starting the experiments at post-natal day 21. The animals were kept in an animal house at a constant temperature of 20 °C with a 12 h alternating light–dark cycle to minimize circadian variations. Before starting the experiment, the mice were housed in the animal facility for 1 week. All efforts were made to minimize animal suffering and the number of animals used. All the experimental procedures were approved by the Italian Ministry of Health (No. 446/2018-PR–20/06/2018) and followed the National Institutes of Health guide for the care and the use of laboratory animals (NIH Publications No. 8023, revised 1978).

The two groups just described, consisting of 20 BTBR mice and 20 CTR mice, respectively, were further divided into two subgroups, creating four experimental groups of 10 mice each. Randomly, one group of 10 BTBR mice and one group of 10 CTR mice were selected to be treated with 10 mg/kg/day per *os* of MLT, while the two remaining subgroups were treated daily per *os* with the MLT vehicle.

The MLT treatment followed the procedure reported by Borsani et al. [111]. MLT was given per *os* through gavage (100 μL) in a single daily administration. At the end of the experiments, as reported in our previous study, all the experimental animals were subjected to behavioral tests (marble burying, self-grooming, and reciprocal social interaction tests). BTBR mice showed an increase in stereotyped and repetitive behaviors and a deficit in social interaction with respect to CTR mice, thus confirming that BTBR mice presented typical ASDs behavioral manifestations [111]. The animals of each experimental group were deeply anesthetized (isoflurane 5%), and five mice per group were transcardically perfused with saline, followed by 50 mL of 4% paraformaldehyde in phosphate-buffered saline (0.1 M, pH 7.4). The liver of each experimental animal was carefully removed for the subsequent morphological and immunohistochemical evaluations. The other five animals of each experimental group were euthanized by cervical dislocation, and then the liver was carefully removed and stored at −80 °C for the subsequent Western blotting evaluations.

**Figure 5:**
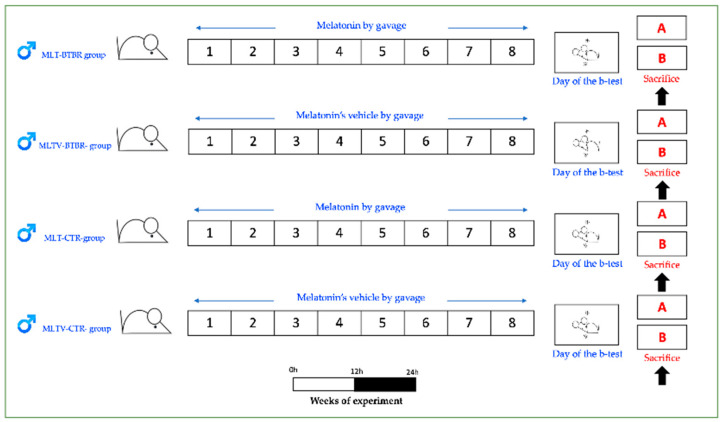
Schematic drawing of the experiment plan. (**A**) indicates the five mice per group transcardically perfused, whereas (**B**) indicates the other five mice per group which were euthanized by cervical dislocation. The black arrows indicate adequate liver samples collection; the blue arrows indicate the experimental treatment duration expressed in number of days (1 to 8); the white box indicates light exposition of mice and the black box indicates the dark exposition of mice. B-test: behavioral test; CTR: control; h: hour; MLT: melatonin; MLTV: melatonin vehicle.

Figure 5 summarized the experimental plan of the present study.

### 4.2. Sample Processing for Morphological and Immunohistochemical Evaluations 

After removal, the liver samples were rinsed in a physiological salt solution, dehydrated in graded ethanol, and embedded in paraffin wax following a standard procedure. Serial paraffin sections (5 μm thick) of each sample were cut with a microtome. 

### 4.3. Mallory–Denk-Bodies-Containing Hepatocytes Evaluation

Alternate liver sections were deparaffinized, rehydrated, and stained with hematoxylin–eosin (Bio Optica, Milan, Italy) according to the standard procedure. The sections were then observed using a light optical microscope (Olympus BX50 microscope, Hamburg, Germany) [3]. A blind examiner identified the hepatocytes with MDBs using the Olympus BX50 microscope at a final magnification of 200×. The number of MDBs-containing hepatocytes was evaluated in 10 liver random fields of each experimental animal, and the resulting data were subjected to statistical analysis.

### 4.4. Immunohistochemical Evaluation 

Alternate liver sections were deparaffinized, rehydrated, and subjected to antigen retrieval in 0.01 M sodium citrate buffer (pH 6.0) in a microwave oven for two cycles of 3 min at 600 W [112]. Then, the sections were washed in tris-buffered saline (TBS) for 5 min and incubated in 3% hydrogen peroxide for 10 min at room temperature. To demonstrate the specificity of antibodies, we used a pre-absorption test (blocking agent): 1% bovine serum albumin in 0.05% Tween 20 for 1 h at room temperature [19]. After that, liver sections were incubated for 45 min at 37 °C followed by 1 h at room temperature with the below primary antibodies: rabbit polyclonal anti-ACSL4 (diluted 1:200; Proteintech Ferroptosis Expanded Antibody Kit, Manchester, UK), mouse monoclonal anti-SQSTM1/p62 (diluted 1:100; Santa Cruz Biotechnology, Dallas, TX, USA), rabbit polyclonal anti-LC3B (diluted 1:200; Invitrogen, Waltham, MA, USA), and rabbit polyclonal anti-NCOA4 (diluted 1:200; Invitrogen, Waltham, MA, USA). Subsequently, the samples were incubated for 1 h with specific biotinylated secondary antibodies (Vector Laboratories, Newark, CA, USA) and then conjugated with an avidin–biotin peroxidase complex (Vector Laboratories, Newark, CA, USA). The reaction products were visualized using 0.33% hydrogen peroxide and 0.05% 3,3′-diaminobenzidine tetrahydrochloride (DAB) as a chromogen (Sigma, St. Louis, MO, USA). Liver sections were counterstained with Carazzi’s Emallumen (Bio Optica, Milan, Italy), dehydrated, mounted and observed with a light optical microscope (Olympus BX50 microscope, Hamburg, Germany) at a final magnification of 400× [113,114]. Immunohistochemical negative controls were performed without the primary antibody but in the presence of the isotype-matched IgG. 

### 4.5. Semiquantitative Analysis

The immunostained sections of each primary antibody investigated were observed with a light optical microscope (Olympus BX50 microscope, Hamburg, Germany) at a final magnification of 200×. Five random fields of a total of two nonconsecutive sections for each liver sample were analyzed by a blind examiner, and the immunostaining for each primary antibody was evaluated. The immunostaining was expressed as negative (−), very weak (±), weak (+), moderate (++), and strong (+++) positivity [115], and the data reported represented the global assessment of the observations made.

### 4.6. Western Blotting Evaluations

Liver lysates were centrifuged at 13,000× *g* for 10 min, and the suspension was collected and stored at −20 °C. Protein concentration was determined using a Bradford assay. Equal amounts of proteins were loaded into 12% SDS polyacrylamide gels and subjected to electrophoresis. The separated proteins were transferred to nitrocellulose membranes, and then the membranes were blocked with 5% bovine serum albumin solution for 1 h at room temperature, followed by overnight incubation in shaking at 4 °C with the following primary antibodies: mouse monoclonal anti-SQSTM1/p62 (diluted 1:300; Santa Cruz Biotechnology, Dallas, TX, USA), rabbit polyclonal anti-LC3B (diluted 1:500; Invitrogen, Waltham, MA, USA), and mouse monoclonal β-actin antibody (diluted 1:2500; Sigma-Aldrich, St. Louis, MO, USA). After washing with tris-buffered saline w/tween-20, the membranes were incubated 1 h at room temperature with an infrared fluorescent-dye-conjugated antibody absorbing at 680 nm. The Western blotting evaluation for each primary antibody investigated was performed in duplicate. The blots were visualized using the Odyssey Fc Imaging System (LI-COR Inc., Bioscience, Lincoln, NE, USA) [19,113]. Quantitative analysis of the bands was performed using ImageJ software (v 1.54g blunded with Java 8—accessed on 2 October 2024) and the resulting data were subjected to statistical analysis.

### 4.7. Statistical Analysis 

Results were expressed as the mean ± standard deviation. Statistical significance of differences among the experimental groups were analyzed using a one-way analysis of variance (ANOVA one-way test, corrected Bonferroni test), with the significance set up at *p* ≤ 0.05. 

## 5. Conclusions

The results of this study should be interpreted cautiously due to the limitation of using only male mice in the experimental design. Taken together, these data suggest that MLT exerts a protective effect against liver damage in BTBR mice by promoting the “correct degradation of misfolded proteins”, including MDBs and damaged organelles, alleviating the impairment of autophagy and *ferritinophagy*/ferroptosis, and attenuating liver damage. MLT may be capable of inducing a metabolic circle that stimulates and modulates both autophagy via p62/LC3B proteins and *ferritinophagy*/ferroptosis via ACSL4 and NCOA4. However, the mechanism of autophagy-mediated ferroptosis remains largely unknown, as reported by Kang and Tang [116], and it is an important area for future studies. 

## Figures and Tables

**Table 1:** Hepatic p62 and LC3B semiquantitative analyses. The immunopositivity was expressed as absent (-), very weak (±), weak (+), moderate (++), and strong (+++).

	*p62*			*LC3B*		
	BTBR	MLT-BTBR	CTR	BTBR	MLT-BTBR	CTR
Cytoplasm	±/+	++	++	+	++	++
MBDs	±/+	±/-	±/-	±	±	±
Nuclei	-	±/-	±/-	++/+++	±	±

**Table 2:** Hepatic NCOA4 and ACSL4 semiquantitative analyses. The immunopositivity was expressed as absent (-), very weak (±), weak (+), moderate (++), and strong (+++).

	*NCOA4*			*ACSL4*		
	BTBR	MLT-BTBR	CTR	BTBR	MLT-BTBR	CTR
Cytoplasm	++	+/±	±	+++	+/++	±
MBDs	±/+	-	-	+/++	±/-	-
Nuclei	++	±	-	+++	-	-

## Data Availability

The original contributions presented in the study are included in the article, further inquiries can be directed to the corresponding author.
